# Clinical Nurse Educators’ Job Satisfaction and Turnover Intentions: Protocol for a Scoping Review

**DOI:** 10.2196/66712

**Published:** 2025-06-20

**Authors:** Kateryna Metersky, Emily Richard, Yasin Yasin, Areej Al-Hamad, Valerie Tan

**Affiliations:** 1 Daphne Cockwell School of Nursing Faculty of Community Services Toronto Metropolitan University Toronto, ON Canada; 2 Department of Nursing Faculty of Nursing University of New Brunswick Moncton, NB Canada; 3 Department of Nursing Faculty of Nursing University of New Brunswick Fredericton, NB Canada

**Keywords:** clinical nurse educators, job satisfaction, turnover intentions, scoping review, acute care hospitals, nurses, acute care, protocol

## Abstract

**Background:**

Clinical Nurse Educators (CNEs) play a critical role in supporting the recruitment, retention, and professional development of staff nurses in acute care hospital settings. In the context of the COVID-19 pandemic, which has exacerbated global nursing shortages, CNEs are reporting high levels of burnout associated with unpredictable work expectations and nursing workforce challenges. However, the factors that influence job satisfaction and turnover intentions among CNEs are not well understood.

**Objective:**

The objective of this scoping review is to provide a comprehensive overview of existing literature that focuses on job satisfaction and turnover intentions among CNEs within the acute care setting.

**Methods:**

Joanna Briggs Institute and PRISMA-ScR (Preferred Reporting Items for Systematic Reviews and Meta-Analyses extension for Scoping Reviews) guidelines will be used for this scoping review. Using a predetermined query of keywords, a comprehensive search will be conducted in SCOPUS, CINAHL, MEDLINE, PsycINFO, Web of Science, citation searching, and gray literature sources to identify studies published between January 2013 and August 2024. This review will consider all literature sources that explore the concept of job satisfaction and turnover intentions of CNEs in the context of acute care settings such as hospitals and short-stay units. Two independent reviewers will screen and select studies, and data extraction will be completed using Covidence (Veritas Health Innovation) systematic review software to gather specific data from each literature source. The results will be synthesized to map out themes within existing literature and to identify areas for further research. Supported by tables and graphs, thematic content analysis will be used to provide an extensive overview of what is known about the topic of interest.

**Results:**

The online database literature search was initiated in January 2025, with a total of 4306 records identified during the initial search. After removing duplicates, 2354 unique records will be screened based on title and abstract. As of February 2025, title and abstract screening is currently underway, with a subsequent full-text screening and gray literature search to follow. We expect data analysis to be completed in April 2025 and results published in May 2025.

**Conclusions:**

Rather than evaluating the significance of findings in individual studies, this review will contribute new insights to the existing literature and identify areas for further research.

**Trial Registration:**

OSF Registries osf.io/ectu7; https://osf.io/ectu7

**International Registered Report Identifier (IRRID):**

DERR1-10.2196/66712

## Introduction

The clinical nurse educator (CNE) is an essential bridge between theoretical knowledge and safe clinical practice in acute care environments. Drawing upon advanced knowledge and experience, CNEs are registered nurses who support staff nurses in upholding patient care quality standards through education and professional development initiatives [1,2]. CNEs also respond to the learning needs of undergraduate nursing students, collaborate with other health care professions, and facilitate the adoption of current evidence-based practices [1,3]. The CNE role has been positively associated with higher levels of work engagement, psychological empowerment, and structurally empowering work environments in acute care hospitals [4].

Against the backdrop of a longstanding global nursing shortage, exacerbated further by the COVID-19 pandemic, the need for strategic actions to attract and retain skilled nursing staff is paramount [5]. CNEs play a critical role in recruiting, training, and advocating for new graduate nurses, many of whom experienced serious transition shock and considered leaving the nursing profession entirely during the COVID-19 pandemic [6,7]. Further, new graduate nurses’ sense of professional and personal identity, as well as the degree of support received, can influence the quality of patient care delivered in hospitals [8]. CNEs have been studied as a protective factor in promoting retention of new graduate nurses in acute care settings; a mixed methods study of novice nurses in Australia suggests that CNE presence aids in developing confidence and resilience, and contributes to the decision to remain active in the nursing profession [9].

However, recent studies conducted in New Brunswick and Ontario have indicated a shortage of experienced nurses equipped with the knowledge and skills needed to mentor early-career nurses entering the workforce [10,11]. Working independently to balance the needs of frontline staff and managerial expectations, the unique role of the CNE has been described as both isolating and overwhelming [12]. Unlike research involving the general nursing population, proposed reasons for job dissatisfaction within the CNE role include lack of role clarity, insufficient support to balance multiple priorities, lack of orientation or preparation to assume the CNE position, and intrinsic pressure to succeed in demanding environments [12]. Anecdotally, CNEs also cite high levels of job dissatisfaction related to heavy workload expectations and frequent turnover of staff nurses, resulting in frequent cycles of new staff orientation, as factors leading to burnout.

While several review papers focus on the factors associated with nurses’ job satisfaction and turnover intention amongst both novice and experienced staff nurses within hospital settings [13-15], there is a paucity of reviews that focus on CNEs in the same regard. Considering the value of the CNE role in supporting positive clinical outcomes for patients and providing transition support for new graduate nurses as well as other newly employed nurses [16,17], it is critical to understand job satisfaction and turnover intentions for this specific population. Findings from this study will provide current insights into clinical practice improvement and human resource management in acute care. This study also establishes a foundation for future research to examine job satisfaction and retention strategies for CNEs in the hospital setting. The research question guiding this review is as follows: What is known in the literature about acute care CNEs’ job satisfaction and turnover intentions?

## Methods

### Inclusion and Exclusion Criteria

This scoping review will follow Joanna Briggs Institute (JBI) recommendations to consider the “PCC” mnemonic, whereby the letters in the acronym stand for Population, Concept, and Context, respectively, to specify which sources will be considered for inclusion [18] and provide a thick description for transferability of study results.

#### Participants

This review will consider studies about CNEs working in acute care hospitals. Recognizing that there are diverse terms used to describe the role of CNEs in the literature, additional search terms will be used to capture relevant sources, including continuing education nurses, resource nurses, advanced practice nurses, or nurse practitioners.

#### Concept

This review will consider literature that pertains to the job satisfaction and turnover intentions of acute care CNEs. Relevant search terms will include: job satisfaction, work satisfaction, job turnover, career turnover, intent to leave, intent to stay, and retention.

#### Context

This review will consider literature specific to the context of acute care settings, including hospitals, short-stay units, ambulatory care facilities, emergency departments, intensive care, coronary care, cardiology, and many general areas where the patient could become acutely unwell and require stabilization. Studies that include the long-term setting and nonclinical areas will be excluded.

### Types of Sources

This review will consider all studies, published between January 2013 and August 2024, which use a range of research methodologies, including quantitative, qualitative, and mixed methods study designs. Gray literature, reviews, editorial pieces, and opinion papers will also be evaluated for potential inclusion. Studies written in English and published in the international health care management and nursing literature will be considered.

## Methods

### Overview

This scoping review protocol was developed following the PRISMA-P (Preferred Reporting Items for Systematic Reviews and Meta-Analyses Protocols) 2015 checklist for systematic review protocols [19] (see Multimedia Appendix 1). The final scoping review will be conducted in accordance with the JBI methodology [20] and the PRISMA-ScR (Preferred Reporting Items for Systematic Reviews and Meta-Analyses extension for Scoping Reviews). The protocol for this proposed review has been registered with the Open Science Framework. This project is exempt from institutional review board approval requirements.

#### Search Strategy

The bibliographic search strategy (see Multimedia Appendix 2) uses various terms to capture the key roles of the CNE, acknowledging the diverse terminology used to describe CNEs within the health care field. This ensures that the review is as inclusive as possible of the relevant literature. The completed scoping review will include all modified search queries for other interfaces within the appendices.

The following databases will be searched: SCOPUS (via Elsevier interface); CINAHL (via EBSCOhost interface); MEDLINE and PsycINFO (via OVID interface); and Web of Science. The search will also include gray literature sources, including ProQuest (Dissertations and Theses Global), along with wider platforms such as Google and Google Scholar to counter the impact of publication bias commonly seen in academic journals, as well as to enhance the depth of understanding within the study topic of interest. This strategic selection of databases and information sources will ensure a comprehensive sweep of health care–specific and broader academic literature.

This review will also use a 3-step search validation procedure to ensure credibility, as follows.

Within the first stage, the search strategy will be applied to a preselected group of relevant articles from CINAHL, identified during the initial literature review. These articles are known to meet the inclusion criteria for the present scoping review. If the proposed search strategy fails to retrieve these articles, the search terms or overall search strategy will be further refined to capture these critical literature sources. In the second stage of search validation, the reviewers will conduct a manual search of leading academic journals to ensure no significant articles of interest are overlooked. Reviewers will also manually examine the reference lists of articles selected for review to identify any further studies, which may not have been captured during the initial search. Finally, as the research team progresses through the screening and review stages, the relevance of articles yielded from the search will be evaluated on a continual basis; the search strategy will be revised as necessary.

#### Procedure to Contact Authors

The reviewers plan to contact authors for additional information or clarification where necessary. If the need arises, the below-streamlined procedures will be followed: (1) criteria for contact; (2) initial email; (3) follow-up process; and (4) author permission for metadata sharing.

Authors will be contacted if essential data in their study is missing, unclear, or if supplementary or updated information is necessary. For the initial email, the reviewers will write a clear and professional email, outlining the purpose of the present review, the specific information needed, and a reasonable response deadline. Any questions or concerns, which may arise from the corresponding author will also be addressed. In the follow-up process, if there is no response from the author within 2 weeks, the reviewers will send a follow-up reminder email. After a third unanswered attempt, we will consider it a nonresponse, ceasing further contact, and recording this information in a detailed log. Finally, for author permission for metadata sharing, our initial emails will seek author consent to share communication metadata in order to ensure transparency and respect privacy. For consistency and professionalism, we will use standard communication templates from OSF Registries.

#### Results of Contacting Authors

We plan to report the outcomes of our contact for transparency and an enhanced understanding of the comprehensiveness of the present review. The report will detail the number of authors contacted, those who responded, and those who provided extra data or clarification of their study findings (including any relevant data or details not published within their study). This information will be summarized and presented within a table, outlining contact attempts, response types, nonresponses, and any other issues encountered. In addition, this information will be included within the “Methods” and “Discussion” sections of our final report to present any potential biases and a full overview of the review’s scope.

#### Search Expiration and Repetition

The search cutoff date is purposefully scheduled close to the beginning of our review process to ensure the inclusion of the most current and relevant research. However, the adaptability of this protocol is a key feature in enhancing the relevance and currency of the review; the time frame will be viewed as a guiding principle rather than an inflexible rule. The search strategy will be adapted as necessary in response to the available literature. The present protocol will remain flexible in adjusting the frequency of updates in response to the publication rate in the field, reflecting an understanding of the dynamic nature of nursing education research.

#### Study Selection

Upon search completion, records will be compiled and uploaded to Covidence software for automatic removal of duplicate citations and article review. Two independent reviewers will screen each title and abstract based on the identified inclusion criteria, with a subsequent full-text review of relevant sources to follow. Any reasons for excluding sources will be recorded and reported. Any disagreements between the two independent reviewers will be attempted to be resolved through discussion; if an agreement cannot be reached, a third, senior reviewer will be consulted.

#### Data Extraction

Data will be extracted from studies included in the scoping review by two independent reviewers, documenting various aspects including study details, such as title, authors, year of publication, country of origin of the research team, journal name (if applicable), and funding sources (if mentioned; see Multimedia Appendix 3). The data will include specific information regarding study design, type of study, and specifics including the setting and duration of the research, population studied, sample size, demographic and professional characteristics of the participants, as well as any inclusion or exclusion criteria applied during the assessment of literature. The concept of the study, focused on job satisfaction and turnover intentions among CNEs, will also be recorded, with specific attention paid to how the acute care context impacts the findings.

#### Data Analysis and Presentation

To ensure a robust analysis, the research team has collaboratively developed and revised the present review protocol and process, led by an experienced member. The reviewers will systematically organize the extracted data pertaining to key themes, author findings, research methodologies, and the context of acute care practice. Next, a thematic analysis will be performed to uncover key themes, patterns, and trends, involving coding of data, theme development, and further refinement. Braun and Clarke’s 6-phase framework [21,22] will be used to conduct thematic analysis, in conjunction with updated guidance from the authors regarding contextual consideration of ideas, flexibility in the development of themes, and documenting ongoing reflection and transparency throughout the process to ensure appropriate reflexivity and confirmability.

Finally, tables and charts (see Multimedia Appendix 4) will be used to create a visual depiction of key findings, including the distribution of literature by publication year, country of origin, research methods, and factors that influence job satisfaction and turnover intention. These visual representations will be complemented with a descriptive summary, which links the results back to the research objectives and questions. In the case that any challenges are encountered during the process (such as data unavailability), the synthesis approach will be adapted, maintaining transparency and providing rationales for any methodological adjustments.

## Results

**Figure 1:**
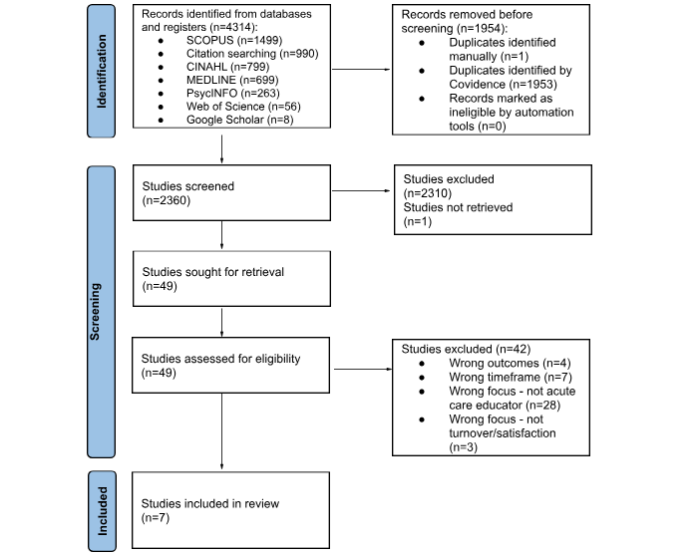
PRISMA (Preferred Reporting Items for Systematic Reviews and Meta-Analyses) flow diagram for the study selection process.

The initial literature search was initiated in January 2025 across the following databases: SCOPUS, CINAHL, MEDLINE, PsycINFO, Web of Science, with additional backward citation searching (reference lists of identified studies were examined to find additional relevant articles for inclusion). A total of 4314 records were identified during the initial search. After removing duplicates, 2360 unique records were screened for inclusion by two independent reviewers based on title and abstract. The PRISMA-*P* 2020 flow diagram [23] was adapted to illustrate the study selection process in Figure 1.

Full-text screening was completed in March 2025. As of May 2025, data analysis is currently underway with results expected to be published in July 2025.

## Discussion

### Anticipated Findings

Recent literature on nursing educators in academic settings suggests that manageable workloads and possessing advanced qualifications is positively associated with job satisfaction, while high workloads are associated with high turnover intentions [23]. We anticipate that the main findings of this study will reinforce what is currently known about job satisfaction in CNEs, namely role confusion, lack of support, overwhelming workloads, and intrinsic pressure to sustain high level performance [13]. Due to high turnover rates of new graduate nurses and experienced staff nurses [14-16] in current times, we also expect to find an equivalent correlation in the studied CNE population.

The findings and conclusions from this study will be impactful to current and prospective CNEs, who may be facing similar challenges and feelings of isolation in the workplace. Staff nurses, nursing management, allied health practitioners, and institutional leadership who work with CNEs can benefit from a more fulsome understanding of the factors which influence job satisfaction and turnover intentions in CNEs in order to improve collaborative workplace culture and subsequently, enhance patient care outcomes.

This study has several anticipated limitations. Since the aim of this scoping review is to explore the extent of research related to CNEs’ job satisfaction, turnover intentions, and work lives, the quality or validity of individual studies will not be evaluated as this is not a requirement in the scoping review process. This may result in the inclusion of lower-quality research or studies with inherent bias. Another limitation of this study is that the findings may not fully reflect current challenges in acute care settings during, and post–COVID-19 pandemic. We plan to mitigate the impact of these limitations by examining diverse research methods and information sources in order to contribute new insights to the existing literature and identify areas for further research.

### Conclusion

This scoping review will explore the extent of research related to CNEs’ work life, job satisfaction, and turnover intention, rather than evaluating the significance or magnitude of findings in individual studies. Therefore, the conclusions of this review will reflect the comprehensiveness and scope of the included studies as a whole to identify and highlight gaps in existing research and suggest potential areas for future study.

## Appendix

Multimedia Appendix 1 PRISMA (Preferred Reporting Items for Systematic Reviews and Meta-Analyses) checklist.

Multimedia Appendix 2 Search query string.

Multimedia Appendix 3 Data Extraction Tool/Instrument.

Multimedia Appendix 4 Table of key findings.

## Abbreviations

CNE: Clinical Nurse Educator
JBI: Joanna Briggs Institute
PRISMA-P: Preferred Reporting Items for Systematic Reviews and Meta-Analyses Protocols
PRISMA-ScR: Preferred Reporting Items for Systematic Reviews and Meta-Analyses extension for Scoping Reviews

## Data Availability

Supporting data will be available upon reasonable request from the corresponding author.
