# Supplementary material for: Clinical Nurse Educators’ Job Satisfaction and Turnover Intentions: Protocol for a Scoping Review
Source: JMIR Res Protoc. 2025 Jun 20;14:e66712. doi: 10.2196/66712 (PMC12228001; doi:10.2196/66712)
Supplement: Multimedia Appendix 2 [file resprot_v14i1e66712_app2.docx]

**Appendix 2: Search query string**

The terms “job satisfaction” or turnover or retention or quitting or “work satisfaction” or “employee satisfaction” or “intention to stay” or “intention to leave” or “quality of work life” or “personnel retention” were used, combined with "nurse educator*" or cne or "continuing education nurse" or "advanced practice nurse" or “nurse practitioner*” or “nursing professional development practitioner*” or “staff development specialist*” or “staff development instructor*” or “staff development educator*” or “professional development nurse*” or “resource nurse*”.
