# Supplementary material for: Clinical Nurse Educators’ Job Satisfaction and Turnover Intentions: Protocol for a Scoping Review
Source: JMIR Res Protoc. 2025 Jun 20;14:e66712. doi: 10.2196/66712 (PMC12228001; doi:10.2196/66712)
Supplement: Multimedia Appendix 3 [file resprot_v14i1e66712_app3.docx]

**Appendix 3: Data Extraction Tool/Instrument**

**Peer-reviewed Literature**

| Author (Year) | Country | Methodology/Methods | Aim/Objective | Target Audience | Findings |
| --- | --- | --- | --- | --- | --- |

**Grey Literature**

| Author | Organization/ Institution | Approach | Focus | Target Audience | Key Findings |
| --- | --- | --- | --- | --- | --- |
