# Supplementary material for: Clinical Nurse Educators’ Job Satisfaction and Turnover Intentions: Protocol for a Scoping Review
Source: JMIR Res Protoc. 2025 Jun 20;14:e66712. doi: 10.2196/66712 (PMC12228001; doi:10.2196/66712)
Supplement: Multimedia Appendix 4 [file resprot_v14i1e66712_app4.docx]

**Appendix 4: Table of Key Findings**

| Author, Year, Country | Methodology/Methods | Aim/Objective | Setting | Relevance/Findings applicable to research question |
| --- | --- | --- | --- | --- |
